# The Ancestral Carnivore Karyotype As Substantiated by Comparative Chromosome Painting of Three Pinnipeds, the Walrus, the Steller Sea Lion and the Baikal Seal (Pinnipedia, Carnivora)

**DOI:** 10.1371/journal.pone.0147647

**Published:** 2016-01-28

**Authors:** Violetta R. Beklemisheva, Polina L. Perelman, Natalya A. Lemskaya, Anastasia I. Kulemzina, Anastasia A. Proskuryakova, Vladimir N. Burkanov, Alexander S. Graphodatsky

**Affiliations:** 1 Department of Comparative Genomics, Institute of Molecular and Cellular Biology, Siberian Branch of Russian Academy of Sciences, Novosibirsk, Russia; 2 Department of Higher Vertebrates Ecology, Kamchatka Branch of Pacific Geographical Institute of Far East Branch of Russian Academy of Sciences, Petropavlovsk-Kamchatski, Russia; 3 National Marine Mammal Laboratory, Alaska Fisheries Science Centre, National Marine Fisheries Service, Seattle, Washington, United States of America; 4 Novosibirsk State University, Novosibirsk, Russia; Texas A&M University, UNITED STATES

## Abstract

Karyotype evolution in Carnivora is thoroughly studied by classical and molecular cytogenetics and supplemented by reconstructions of Ancestral Carnivora Karyotype (ACK). However chromosome painting information from two pinniped families (Odobenidae and Otariidae) is noticeably missing. We report on the construction of the comparative chromosome map for species from each of the three pinniped families: the walrus (*Odobenus rosmarus*, Odobenidae–monotypic family), near threatened Steller sea lion (*Eumetopias jubatus*, Otariidae) and the endemic Baikal seal (*Pusa sibirica*, Phocidae) using combination of human, domestic dog and stone marten whole-chromosome painting probes. The earliest karyological studies of Pinnipedia showed that pinnipeds were characterized by a pronounced karyological conservatism that is confirmed here with species from Phocidae, Otariidae and Odobenidae sharing same low number of conserved human autosomal segments (32). Chromosome painting in Pinnipedia and comparison with non-pinniped carnivore karyotypes provide strong support for refined structure of ACK with 2n = 38. Constructed comparative chromosome maps show that pinniped karyotype evolution was characterized by few tandem fusions, seemingly absent inversions and slow rate of genome rearrangements (less then one rearrangement per 10 million years). Integrative comparative analyses with published chromosome painting of *Phoca vitulina* revealed common cytogenetic signature for Phoca/Pusa branch and supports Phocidae and Otaroidea (Otariidae/Odobenidae) as sister groups. We revealed rearrangements specific for walrus karyotype and found the chromosomal signature linking together families Otariidae and Odobenidae. The Steller sea lion karyotype is the most conserved among three studied species and differs from the ACK by single fusion. The study underlined the strikingly slow karyotype evolution of the Pinnipedia in general and the Otariidae in particular.

## Introduction

Pinnipeds are remarkable group of marine animals with unique adaptations to semi aquatic life comprising seals, sea lions and walrus. The pinnipeds were one of the first mammalian species group to be thoroughly investigated at each stage of cytogenetics development. Animal cytogenetics made great progress over the past century starting with introduction of techniques for efficient chromosome visualization (like colchicine application, hypotonic treatment, advances in cell culture and chromosome preparation) allowing detailed description of karyotype [1]. Development of differential staining methods (G-banding in particular) allowed identification of homologous elements and chromosomal rearrangements in karyotypes of different species. This enabled karyotype comparison of different mammalian species and gave rise to comparative mammalian genomics producing vast data about chromosome evolution. The introduction of chromosome specific probes ensured precise delineation of homologous syntenic regions and identification of evolutionary chromosome rearrangements even among distant species.

The question about the role of chromosome rearrangements during speciation remains one of the few unsolved and intriguing puzzles of current evolutionary biology. Even more interesting are the genome-shaping evolutionary processes of speciation in marine environment. Fin-footed pinnipeds have split from terrestrial members of Carnivora about 40 MYA [2]. This group is so distinct that they were once considered to be a separate suborder of Carnivora or separate mammalian order Pinnipedia [3,4]. There was even question whether pinnipeds are monophyletic or the true seals had originated from weasels and the other two families from bears [5]. In recent decades molecular phylogenetic studies have settled many questions showing that Pinnipeds are monophyletic and affine to Caniformia branch of Carnivora with some remaining uncertainty about grouping with musteloids or ursids [2,3,6,7].

The Pinnipedia include three extant families, Odobenidae (walruses, monotypic), Otariidae (sea lions and fur seals) and Phocidae (true or earless seals). Odobenidae and Otariidae constitute the Otaroidea, the sister group of Phocidae. The pioneering karyological studies of Pinnipedia and later comparisons based on differential staining techniques showed that pinnipeds have narrow range of diploid numbers 2n = 32 to 36 and are characterized by a pronounced karyological conservatism [8–12]. All Otariidae have strikingly similar karyotypes with the chromosome number 2n = 36. The chromosome number of the walrus is 2n = 32. The Phocidae have two chromosome numbers, 2n = 34 and 2n = 32, separated by a single fusion [8].

Genomes of Carnivora have been investigated in detail both by classic and by molecular cytogenetic methods that showed trends of karyotype evolution in two branches of this order—Caniformia and Feliformia. To date 50 species of Carnivora have been studied by fluorescent *in situ* hybridization (FISH) with flow-sorted whole-chromosome probes from nine Carnivora species and human [13,14]. The conserved syntenic segments and rearrangements of these blocks were established for most families depicting fascinating karyotypic changes during Carnivora radiation. Two major patterns of genome evolution were revealed for this order: fast genome reshuffling during speciation in Canidae, Ursidae and Mephitidae and significant level of conservation among felids, viverrids, and musteloids (Mustelidae, Procyonidae) [13,15–25].

Comparative cytogenetics of Carnivora provided the basis for reconstruction of the karyotype of the supposed common ancestor (Ancestral Carnivora Karyotype, ACK) [19,26–29]. In the original attempt to reconstruct ACK based on comparative G-banding analysis pinniped karyotypes played an important role [26]. The first hypothetical structure of ACK using chromosome painting was based on Pinniped data [28]. However current versions of reconstructed ACK contain some unsettled questions concerning the state of several ancestral segments that require additional support.

To date sequencing data for ten Carnivora species: cat, tiger, lion, giant panda, domestic dog, polar and brown bears, ferret and two pinnipeds—walrus and Weddel’s seal, have been published (http://www.ensembl.org, http://www.ncbi.nlm.nih.gov/genome). Sequencing of the Baikal seal genome is in progress (unpublished). Comparative chromosome painting maps provide primary backbone for *de novo* whole genome assembly. Yet high-resolution G-banded chromosomes and molecular assignment of homologies through chromosome painting is only available for one pinniped species—the harbour seal (*Phoca vitulina*) that belongs to family Phocidae [28,30]. However two other families of pinnipeds (Otariidae and Odobenidae) are lacking the chromosome painting data [13].

Here, we report comparative chromosome maps for representatives from all three pinniped families: the walrus (*Odobenus rosmarus*, Odobenidae), Steller sea lion (*Eumetopias jubatus*, Otariidae) and the Baikal seal (*Pusa sibirica*, Phocidae) established by localization of human, domestic dog and selected set of stone marten chromosome-specific painting probes, compiled with previously published chromosome painting data. We conduct analysis in the context of chromosome evolution in the order Carnivora and in eutherian mammals and refine the ACK based on the new evidence from Pinnipedia chromosome painting.

## Materials and Methods

### Species sampled

We used samples from wild animals. Tissues from the walrus were collected during aboriginal quota sealing in the coastal waters of Bering Sea (Mechigmen bay, Chukotka Autonomous Okrug, Russian Federation). Flapper and ear skin biopsy samples from Steller sea lions were collected on the Tyuleniy Island (the Sea of Okhotsk, Russia) when pup’s were branded. Isoflurane was used for inhalational anaesthesia. Tissue samples of the Baikal seal (Lake Baikal, Barguzin bay, Russian Federation) were taken from the male killed by fishnets and reported for the scientific research. All samples were collected according to procedures approved by the Committee on the Ethics of Animal Experiments of the Institute of Molecular and Cellular Biology SB RAS. The information about species used in this study is listed in Table 1.

**Table 1:** 

No	Latin names	Codes	2N	Sex	Common names
1	*Odobenus rosmarus*	OROS	32	M	Walrus
2	*Eumetopias jubatus*	EJUB	36	M	Northern sea lion
3	*Pusa sibirica*	PSIB	32	M	Baikal seal
4	*Canis familiaris*	CFA	78	M	Domestic dog
5	*Martes foina*	MFO	38	M	Stone marten
6	*Homo sapiens*	HSA	46	M	Human

### Storage and transportation of tissue samples

The lack of cytogenetic data for wild marine mammals and the limited number of available primary cell lines may be explained in part by the remoteness of their habitats and so by difficulties of collecting and delivering aseptic viable tissue samples to the laboratories. While it took 10–14 days to transport specimens to the laboratory from remote areas of collection additional efforts to prevent bacterial and mycotic contamination were undertaken. Upon collection all tissue pieces were thoroughly washed and scrubbed in continuous cool water flow to remove dirt and sand. Then samples were incubated for 20–30 min in growth medium with 5 times excess of antibiotic mixture (gentamicin sulphate+ampicillin+amphotericin b), followed by the incubation in medium with 3 times excess of antibiotics. After the antibiotic/antimycotic treatment all samples were placed into individual 5ml plastic tubes containing sterile transporting medium (growth medium αMEM (Gibco) with 15% bovine calf serum (HyClone), gentamicin sulphate 50mg/L, ampicillin 100 mg/L and amphotericin b 2.5mg/L). Forceps and scissors were washed in soapy water, rinsed in water, then soaked in 96% ethanol and flame-sterilised over the alcohol burner before manipulating each sample. Tubes with such processed samples were kept in the refrigerator at a temperature +4–10°C. Every time (approximately once in two or three day) when the medium in tubes was acidifying and changing the colour samples were moved into new sterile tubes containing fresh transporting medium using aseptic precautions and flame-sterilized tools.

### Chromosome preparation and banding techniques, chromosome nomenclature

The primary fibroblast cell lines were derived from biopsies of ear, lung, sinew or flapper tissues of *O*. *rosmarus* (OROS), *E*. *jubatus* (EJUB) and *P*. *sibirica* (PSIB) using conventional techniques [31]. Metaphase preparations were made as described earlier [22,32,33]. Standard G-banding staining was performed according to [34] method. Chromosomes of OROS, EJUB and PSIB were arranged by length.

### Chromosome-specific painting probes preparation and characterization

Sets of human, domestic dog and stone marten chromosome-specific painting probes were described previously [22,35,36]. In this investigation we used the dog chromosomal nomenclatures published by [22]. Whole-chromosome dog library was used for FISH on *O*. *rosmarus* (OROS), *E*. *jubatus* (EJUB) and *P*. *sibirica* (PSIB) genomes. Painting with human probes was done on EJUB and PSIB chromosomes.

### Image capture and data processing

Digital images of the hybridization signals were captured as described by [22,33,36] using the VideoTest system (St.- Petersburg) with a CCD camera (Jenoptic) mounted on a Zeiss microscope Axioscope 2. Metaphase spreads images were edited in Corel Paint Shop Pro Photo X2.

## Results

### Hybridization of dog probes onto chromosomes of three pinniped species

**Fig 1:**
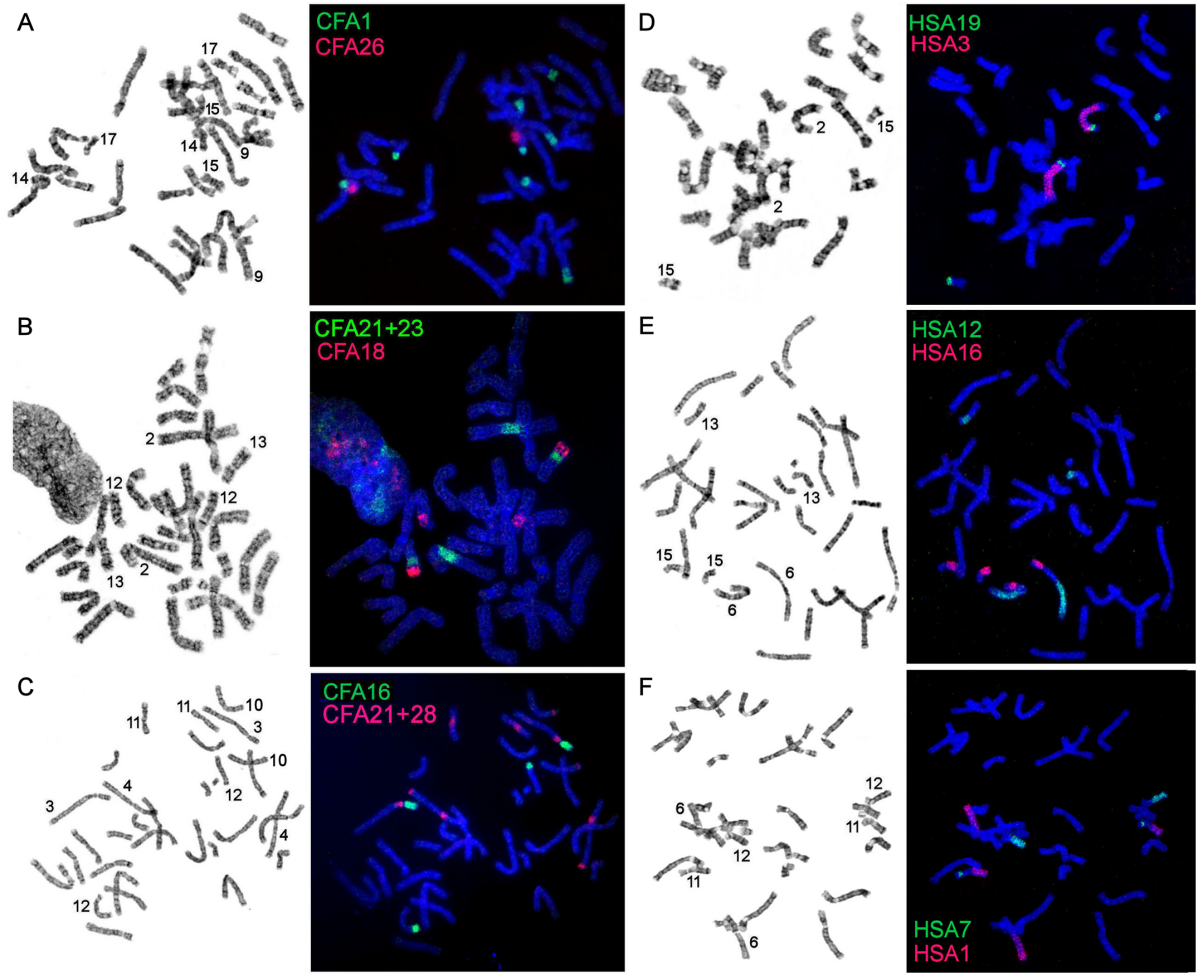
Examples of localization of dog (A-C) and human (D-F) painting probes with GTG-banding of the same metaphase to the right. (A) CFA1/26 on EJUB chromosomes; (B) CFA 21+23/18 on OROS chromosomes; (C) CFA16/21+28 on PSIB chromosomes; (D) HSA 19/3 on OROS chromosomes; (E) HSA12/16 on PSIB chromosomes; (F) HSA7/1 on OROS chromosomes.

**Fig 2:**
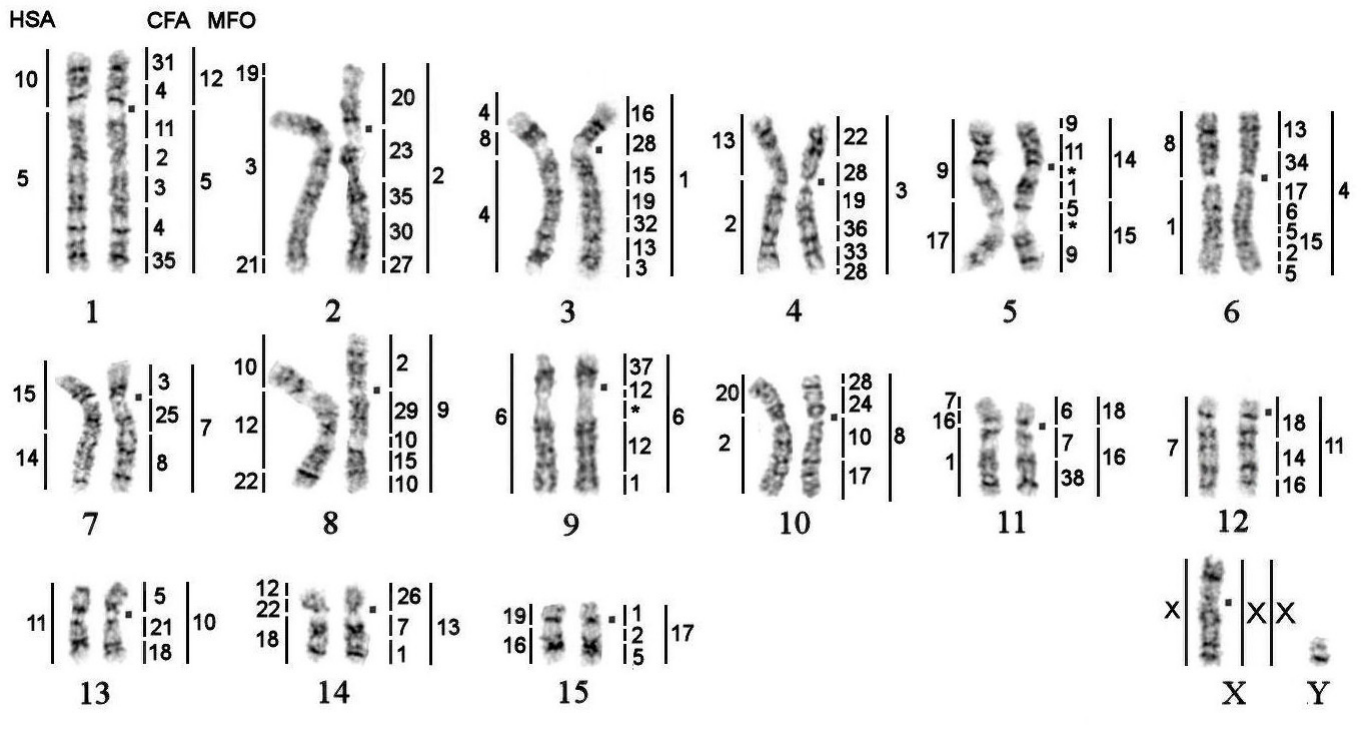
GTG-banded karyotype of walrus (*Odobenus rosmarus*, 2n = 32) with the assignment of homology to human (HSA), dog (CFA) and stone marten (MFO) chromosomes. The square denotes the centromere position on corresponding chromosome. *—segments were not painted by any dog probe.

**Fig 3:**
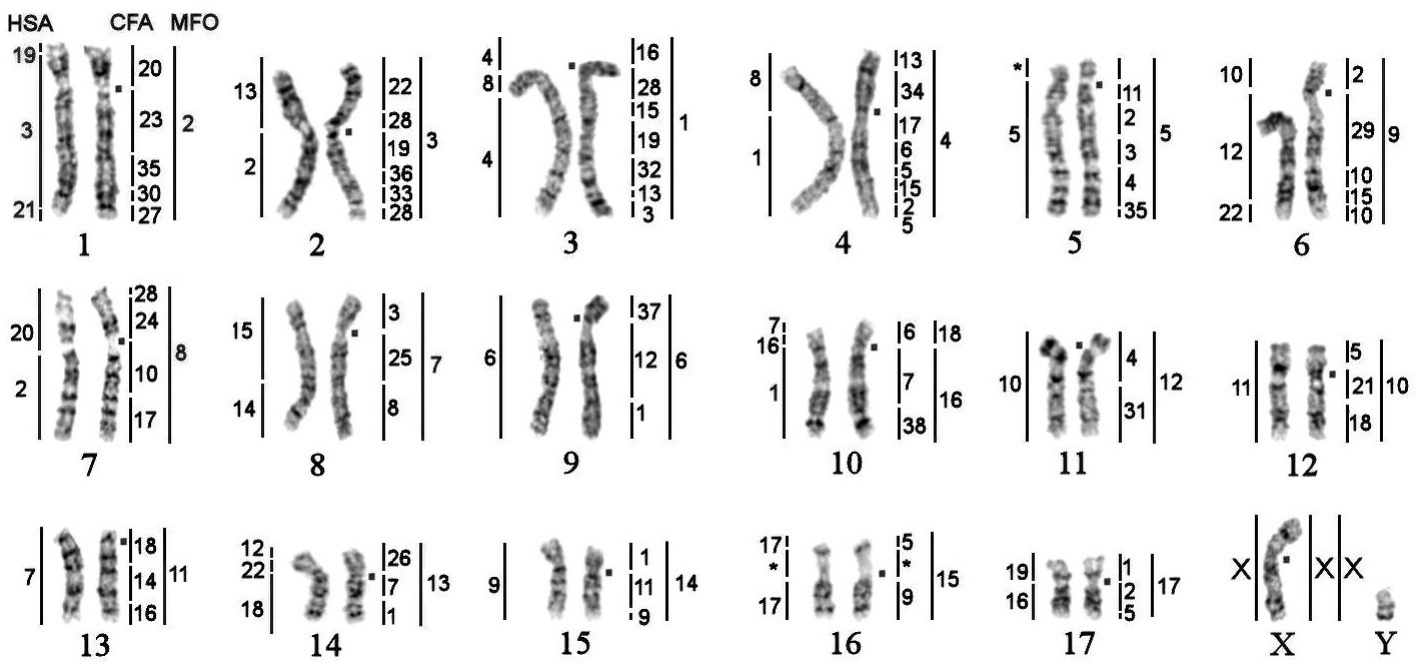
GTG-banded karyotype of the Steller sea lion (*Eumetopias jubatus*, 2n = 36) with the assignment of homology to human (HSA), dog (CFA) and stone marten (MFO) chromosomes. The square denotes the centromere position on corresponding chromosome. *—segments were not painted by any dog probe.

**Fig 4:**
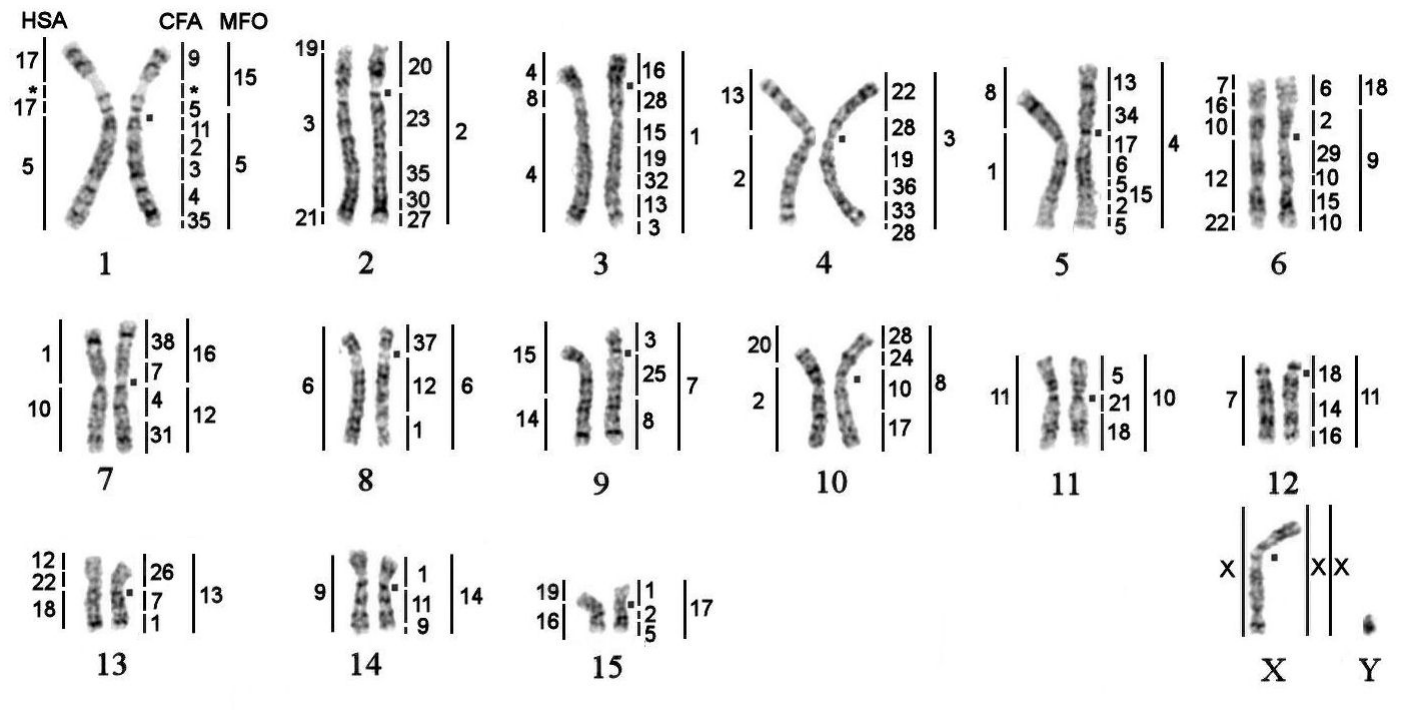
GTG-banded karyotype of the Baikal seal (*Pusa sibirica*, 2n = 32) with the assignment of homology to human (HSA), dog (CFA) and stone marten (MFO) chromosomes. The square denotes the centromere position on corresponding chromosome. *—segments were not painted by any dog probe.

Hybridization examples of CFA probes are shown in Fig 1A–1C. Each CFA probe highlighted 1 to 5 fragments in the karyotypes of walrus, Steller sea lion and the Baikal seal. The CFA autosomal probes detected 68 homologous chromosomal segments in the genomes of investigated pinniped species. Twelve autosomes and X-chromosome are conserved *in toto* in the studied karyotypes. Chromosome maps of OROS, EJUB and PSIB are presented in Figs 2, 3 and 4 respectively. The homology to chromosomal segments of MFO is based on a published comparative chromosome data between MFO and CFA [13] and shown to the right of pinniped chromosomes.

### Mapping of human probes onto chromosomes of the walrus, Steller sea lion and the Baikal seal

The 22 HSA whole autosomal probes revealed 32 homologous chromosomal segments in the genomes of EJUB, OROS and PSIB. Patterns of chromosome painting with human chromosome-specific painting-probes are demonstrated in Fig 1D–1F. Each HSA probe painted 1 or 2 fragments in the karyotypes of studied species. Human chromosome probes HSA 6, 11 and X each delineated a complete chromosome in the walrus, Steller sea lion and the Baikal seal karyotypes. Probes containing HSA 5 and 16 each detected an entire chromosome in the EJUB chromosomal set, HSA 9 painted a whole chromosome in EJUB and PSIB. 10 chromosome breaks, one inversion and 15 (for EJUB) or 16 (for PSIB and OROS) fusions are required to convert the human karyotype into the pinniped karyotypes.

### Mapping of selected stone marten painting probes onto chromosomes of pinniped species

Stone marten chromosomes 15 and 5 were mapped onto metaphases of the Baikal seal, probe combinations of MFO 14 /15 and 12/5 were mapped onto the walrus metaphases to clarify arrangement of ancestral carnivore syntenic elements ambiguous after analyses of human and dog painting probe localizations.

## Discussion

There were many attempts to shift into aquatic or semi-aquatic lifestyle in different mammalian orders—Cetartiodactyla (whales, dolphins, porpoises), Afrotheria (manatee, dugong), Carnivora (pinnipeds, otters, polar bear), Rodentia (beavers, water vole and others). Pinnipeds hunt, sleep and spend most of their life in the water but come out to bear their pups. The question about genetic impact of the unique position of pinnipeds as marine semi-aquatic animals among mostly terrestrial Carnivora is very intriguing. Surprisingly such drastic change in the mode of life is not accompanied by critical changes at the genetic level. Adaptations to aquatic environment are reflected in amino-acid substitutions of positively selected genomic regions found in pinnipeds and convergent with other marine mammals (whale and manatee) and yet accompanied by remarkably low substitution rate in mutation prone CpG islands [37]. There is also indication of low level of variation in skull morphology between pinnipeds and fissipeds [38]. These modern findings reflect on the old hypothesis of chromosomal conservatism in marine mammals [39] that states “speciation caused by chromosomal rearrangements is rare within mammalian orders characterized by low reproduction, good mobility and continuous range of distribution”.

### Chromosome evolution in Pinnipedia

The concept of high degree of chromosome conservation in pinnipeds is based on the classical cytogenetic data that have revealed similar diploid numbers (32–36) across this group of semi-aquatic mammals and showed high similarity of G-banding pattern with other mammals. It is supported by painting findings of the low numbers of conservative ancestral elements in harbour seal compared to other mammals [8–11,28]. Our data provide further support documenting remarkable chromosome conservation across all families of pinnipeds. 22 human autosome painting probes delineated 32 conservative autosome segments in the genomes of three studied species. This corresponds to assessment of their genomes as low-rearranged relatively to the ancestral eutherian genome. Similarly low number of autosome segments was detected by human painting probes in the conservative karyotypes of musteloids, felids, hyenas and some viverrids [13,40,41] and conserved karyotypes of other mammals like aardvark and golden mole (30). Much higher numbers are found in other mammals (47 for closely related pangolins, 40–50 in cetartiodactyls and perissodactyls, 42 in bats) or even highly rearranged Carnivora karyotypes (67–73 in canids, 44 in ursids, 40 in skunks) indicating higher divergence from ancestral eutherian karyotype. Chromosome painting in pinnipeds here confirms long-standing hypothesis about karyotype conservatism in marine animals. The underlying reasons for this phenomenon were discussed and include low degree of inbreeding, low reproductivity rate and absence of physical geographic barriers [39]. Other marine animals with exception of afrotherian manatee (41) also have low number of conservative ancestral units: otter (32), pilot whale (34), finless porpoise (34) and bottlenose dolphin (30). Latter three species belong to the order Cetartiodactyla and at the same time other members of the order have over 45 conservative chromosome segments [13,33,42–44]. Both groups—Cetacea and Pinnipedia are rather specious (with more than 80 and 32 species) demonstrating that conservative karyotype does not impede the speciation and that the species radiation is not always accompanied by apparent chromosome rearrangements.

We revealed in the genomes of three studied pinnipeds conserved syntenic segment associations common for the whole clade Eutheria (HSA 3/21, 4/8, 7/16, 10/12/22, 14/15 and 16/19; [24,29] (Table 2). In total pinniped karyotypes differ from ancestral Eutherian karyotype [45,46] by 5 fusions (HSA1/8, 2/20, 2/13, 19+3/21, 12/22+18)) and one fission (HSA1). Four syntenic segment associations (HSA 1/8, 2/13, 2/20, 19+3/21) are characteristic for the whole order Carnivora with few exceptions [14] and represent elements of ancestral Carnivora karyotype with 2n = 38 [47]. Synteny HSA 12/22/18 was suggested to be a cytogenetic character linking Carnivora and Cetartiodactyla [13,42,44]. It was also suggested that there is an association HSA 1q/10q (with HSA1q being really small size—HSA1q42.1–q43) that is common for all Boreutherian mammals [48]. However we did not see the segment of 1q on the corresponding pinniped chromosomes (OROS1, EJUB11, PSIB7) and it is likely that the size of this HSA1q segment is at the threshold of chromosome painting resolution. There is no common cytogenetic trait to distinguish all pinnipeds from the rest of Carnivora and to provide cytogenetic link for monophyletic origin of this group versus once suggested diphyly [5].

**Table 2:** Correspondence of pinniped (the Steller sea lion, the walrus, the Baikal seal and the common seal^1^) chromosomes with dog, human, ancestral carnivore karyotype (ACK^2^), stone marten^3^ and cat^3^ chromosomes.

EJUB	OROS	PSIB	PVIT	CFA	HSA	ACK	MFO	FCA
1	2	2	sm1	20/23/35/30/27	19p/3/21	1	2	A2p/C2
2	4	4	m1	22/28/19/36/33/28	13/2q	3	3	A1p/C1q
3	3	3	sm2	16/28/15/19/32/13/3	4q/8p/4p+q	2	1	B1
4	6	5	m2	13/34/17/6/5/15/2/5	8q/1p+q	4	4	F2/C1p
5	1q	1q	sq	11/2/3/4/35	5	5	5	A1q
6	8	6pprx	m3pprx	2/29/10/15/10	10p/12/22	8	9	B4
7	10	10	m5p	28/24/10/17	20/2p	9	8	A3
8	7	9	sm4	3/25/8	15/14	7	7	B3
9	9	8	sm3	37/12/1	6	6	6	B2
10p	11p	6pdist	m3pdist	6	7/16p	18	18	E3
10q	11q	7p	m4p	37/8	1q	16	16	F1
11	1p	7q	m4q	4/31	10q	12	12	D2
12	13	11	m6	5/21/18	11	11	10	D1
13	12	12	sm5	18/14/16	7	10	11	A2q
14	14	13	m7	26/7/1	12/22/18	13	13	D3
15	5q	14	m8	1/11/9	9	14	14	D4
16	5p	1p	sp	9/5	17	15	15	E1
17	15	15	m9	1/2/5	19q/16q	17	17	E2
X	X	X	X	X	X	X	X	X

Correspondence with human chromosomes is based on our FISH results, human chromosome segments are designated according to published data:

^1^Frönicke et al. [28],

^2^Perelman et al. [14],

^3^Nie et al. [13]).

Here we use dog chromosome nomenclature according to Yang et al., [22]. dist—distal part of chromosome, prx—proximal part of chromosome.

Baikal seal is endemic species found only in Lake Baikal with its origin still being under discussion. Mitogenomic data indicate that it has separated from other *Pusa* species at least 4 MYA [49]. Previously, only conventionally stained karyotype of *Pusa sibirica* was reported [50]. Here we present G-banded karyotype of *Pusa sibirica* with established homologies to human and dog genomes. Zoo-FISH of harbour seal (*Phoca vitulina*, PVIT) with human whole-chromosome probes disclosed 30 autosomal conserved syntenic segments[28] compared to 32 revealed in *Pusa sibirica*. High similarity of GTG-banding of *P*. *vitulina* [30] and *P*. *sibirica* indicates that two conservative fragments were missed in the first analysis of pinniped by comparative chromosome painting: the distal part of p-arm of PVITm3 is homologous to HSA12 and the distal part of p-arm of PVITm7 is homologous to HSA7. It means that the genome of harbor seal contains syntenies homologous to HSA 7/16 and 12/22+18 and consequently there are 32 autosomal segments homologous to human conservative fragments in the genome of PVIT likewise in the genome of PSIB. Otherwise comparison of G-banding and human probes painting patterns in *Phoca vitulina* and *Pusa sibirica* karyotypes here did not reveal any differences showing high level of karyotype conservation during speciation in Phocinae. That proves that whole-chromosome probes would not be helpful to uncover the history of speciation in Phocinae to solve unsettled question about *Phoca/Pusa* division and indicates the necessity for further phylogenetic studies [51].

Karyotype of the walrus, the only living representative of Odobenidae, was described previously [10]. Attempts to reveal rearrangements between walrus and other pinnipeds did not yield conclusive results[10,52]. Using combination of human and domestic dog painting probes of high power of resolution we revealed two fusions characteristic for Odobenidae: HSA 5/10 and 9/17 (ACK 5/12, 14/15) that had remained unresolved in studies of G-banded karyotypes of Otariidae and Odobenidae. Now with links of the walrus chromosomes to well-annotated human and dog genomes the chromosome assignment of existing walrus genome scaffolds may be deducted to provide valuable linkage information and to track evolutionary changes in chromosomes at the fine sequence level (http://www.ncbi.nlm.nih.gov/genome/14031).

**Fig 5:**
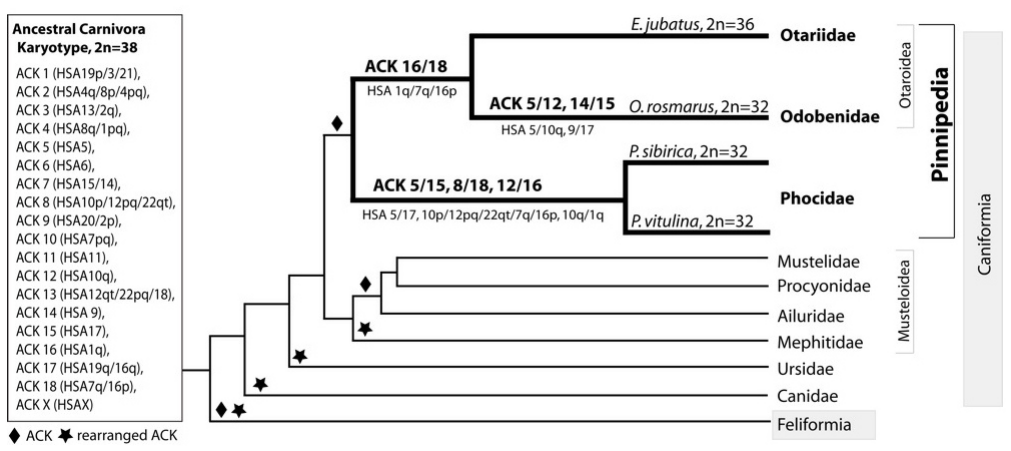
Karyotype evolution pathways in Pinnipedia.

On the base of FISH data with dog painting probes we were able to detect fusions of conservative elements homologous to stone marten autosomes (*Martes foina*, MFO) [13]. So we used some whole chromosome probes of MFO to check these assumptions and to reconstruct ancestral carnivore karyotype reorganization during formation of seal’s genomes. Here we apply the ACK chromosome numbers corresponding to [47] nomenclature. Fusions homologous to HSA 5/17, 7/16+10+12/22 and 10/1 (ACK 5/15, 8/18 and 12/16) found in the Baikal seal genome are common with *Phoca vitulina* and are likely markers for the whole Phoca/Pusa branch. Fusion HSA 7/16+1 (ACK 16/18) is common for Steller sea lion and the walrus. This rearrangement common for monospecific Odobenidae and representative of Otariidae provides strong support for the growing molecular evidence in solving the long-standing puzzle of affinity of Odobenidae with either Otariidae or Phocidae [4]. Incidentally this fusion is also found in bears with exception of giant panda [19,23,24]. However the order of dog chromosome probes is different on this syntenic group (CFA7/38/6 in bears and CFA6/7/38 in walrus and sea lion) and most likely indicates hemiplasic nature of this fusion rather than common chromosomal signature for Arctoidea (Ursidae+Pinnipedia +Musteloidea) or affinity of Ursidae and Pinnipedia [2,6,53,54] (Fig 5). The genome of Steller sea lion retained ancestral state and differs from Ancestral Musteloid Karyotype [33] by only one fusion ACK 16/18 (HSA 7/16+1). This agrees with conclusion of the comparative G-banding analyses that otariid karyotype retained the most primitive features and strikingly resembles conservative procyonid karyotypes [10]. Chromosome painting here precisely showed rearrangements separating pinniped karyotypes erasing uncertainties of sole G-banding comparisons and revealed characteristic chromosomal markers for branching order in Pinnipedia.

### Refinement of Ancestral Carnivore Karyotype by pinniped painting data

First ancestral carnivore karyotype reconstruction was done by Wurster-Hill and Gray based on comparative chromosome analyses of G-banded karyotypes of large number of carnivore species including available data for pinnipeds [9,26]. It was called “hypothetical primitive karyotype” and suggested to contain at least 16 autosomes with minimum fundamental number of 58. It was notable attempt to reconstruct for the first time ancestral karyotype for the whole mammalian order that turned successful owing to remarkably conservative chromosome sets in both carnivore branches of feloids (Felidae, Viverridae) and canoids (Procyonidae, Pinnipedia). Appearance of the gene mapping data for human and cat led to the next variant of ancestral karyotype called CAR that contained 21–22 autosome pairs with FN = 60–68 [27,55] and was importantly shown to be very close to the primate ancestral karyotype. Extensive FISH data provided reliable base for new reconstructions of ACK. Yet lack of painting data for several key branches of Carnivora (Otariidae, Odobenidae, Prionodontidae, Nanndiniidae) contributes to some uncertainties in ACK.

Pinnipedia occupy unique position among canoids: if families with drastically rearranged karyotypes (Canidae, Ursidae, Mephitidae) are not taken into account then pinnipeds represent the most basal canoid branch and become important lineage for ancestral karyotype reconstruction. Based on chromosome painting data in Carnivora two versions of ACK differing by diploid numbers have been proposed: i.e. 2n = 42 [27,29] and 2n = 38 [28,47]. The differences are provided by fused or split status of autosome elements homologous to FCA A1p/ C1q and C1p/F2. Our data demonstrate that in karyotypes of three studied pinniped species these fragments are present as a joint element and this is an argument for the ACK diploid number 2n = 38 as it was first suggested in Z-CAR version of ancestral karyotype based on Phocidae painting. Also all pinnipeds have fused state for FCA A2p/C2, which, in contrast, is present as two separate pieces in all feloid species studied by chromosome painting so far. This fusion is ancestral for the whole order Carnivora.

**Fig 6:**
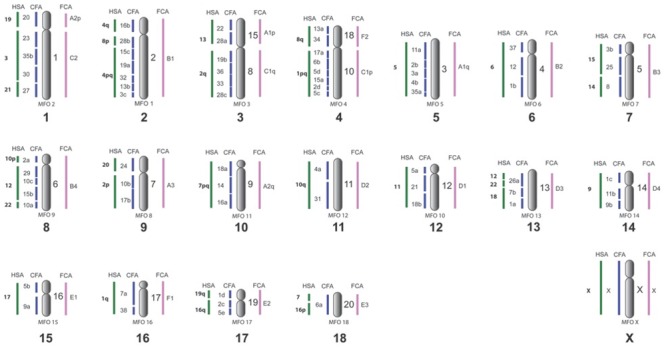
Ancestral carnivore karyotype (ACK). Numbers on the left are HSA and CFA chromosomes. Numbers on the right of each ancestral chromosome are ACK chromosomes according to nomenclature of Murphy et al [26] followed by cat (FCA) homology [13]. MFO chromosome numbers are indicated right below the ancestral chromosome. Big numerals below correspond to Nash et al. [47] ACK nomenclature. The proposed ancestral order of syntenic segments corresponding to dog chromosomes is based on parsimony analyses of chromosome painting data of canid painting probes in Carnivora and two outgroup species: pig and human, [18,19,53]. We exchanged ACK11 and ACK12 relative to ACK published in [11] to match chromosome order [47] and to be arranged according to the chromosome size in cat and stone marten karyotypes.

Refined version of ACK with 2n = 38 that includes painting data of pinniped karyotypes is presented on Fig 6. The mapping of dog painting probes in three pinniped species provides firm evidence toward plausible order of dog chromosome syntenic segments on ancestral chromosomes. The number of conserved segments of dog autosomes is 68. New painting data bring several changes into our previous version of ACK [13,14]. We have found additional fragment corresponding to CFA28 that have not been seen in previous painting studies. In our FISH experiments we used mixed painting probes containing CFA21+28 and CFA21+23. The probe CFA21+23 marked two fragments in the genomes of OROS, EJUB, PSIB, each autosome delineated single segment. The probe containing two dog chromosomes CFA21+28 detected five conservative segments in karyotypes of three studied species: if CFA21 delineates one fragment then CFA28 detects four fragments (Fig 1a). It means that besides three fragments on ACK 2q, ACK 3p and ACK 3q the probe containing CFA28 paints the forth conservative elements on the distal part of OROS 10p, EJUB 7p and PSIB 10p corresponding to ACK 9p dist (CFA28/24/10/17 = HSA20/2p = MFO8 = FCAA3 = ACK9). These chromosomes of the walrus, Steller sea lion and the Baikal seal have very similar banding patterns aligning with homologous chromosomes of cat, ringtail, dwarf mongoose and the Malagasy civet [47]. It is possible that the fourth conservative fragment of CFA28 was not noticed before due to the small size. There is a gap indicated on the comparative map of *H*. *malayanus* (Ursidae) on the chromosome 13 that likely contains this small corresponding fragment homologous to CFA28 [23]. We have verified the presence of this fragment in *Bassariscus astutus* (Procyonidae) by painting of corresponding dog probes and confirmed it. Additional experiments are required to confirm the presence of the fragment in other carnivore families. This additional CFA28 segment, however, it is not reflected in Ensemble synteny data.

The second change in the current ACK structure [14] concerns conservative segment homologous to CFA26. This conservative segment is present in Ensemble synteny data based on whole-genome sequence alignments for HSA 10 but was not detected by painting earlier (http://www.ensembl.org/Canis_familiaris/Location/Synteny?r=26:30309989-30409989). Painting probe containing this autosome detected only one fragment corresponding to ACK13 (HSA 12/22, FCA D3p) in the karyotypes of three studied species and did not reveal the second fragment of CFA26b located on ACK11 (HSA 10, FCA D2p). It remains to be answered why such large fragment (size is about 9 Mb) is not revealed by painting. For now based on the painting data we do not include this segment onto the ACK map.

Based on the analysis of G-banded pinniped karyotypes Nash [52] correctly predicted that fusion FCA F1/D2 (ACK 16/12) is present in Phocidae and absent in Otariidae, and FCA E3 is fused with B4 (ACK 18/8) in Phocidae and with F1 in Otariidae and Odobenidae (ACK 18/16). Our comparison of G-banded chromosomes of OROS, EJUB and PSIB with proposed G-banded ACK [47] confirms that pinniped karyotypes share conservative banding patterns with other carnivore families that retained in whole ancestral karyotype of the order (Fig 6).

Does strict conservation at the level of the chromosome numbers and large chromosome segments in pinnipeds suggest some evolutionary changes at the intrachromosome level? Dog and raccoon dog chromosome painting probes are a good tool for research of intrachromosome structure in Carnivora. Inversions were revealed in many carnivore families studied with canid painting probes [13]. In contrast we did not find any inversions in studied pinniped species. The order of conservative syntenic segments homologous to dog autosomes on chromosomes of the walrus, Steller sea lion and the Baikal seal is identical and corresponds to the suggested sequence of conservative dog elements in the ACK [14]. Thus karyotypic conservation in pinnipeds extends to the intrachromosomal level. The question remains whether the same level of conservation will be seen in comparative analyses of pinniped’s whole genome sequences.

Genomes of Pinnipedia diverged from other Carnivora about 40 MYA with Odobenidae-Otariidae split happening at roughly 25 MYA [49]. During this time only few karyotype rearrangements took place suggesting that the rate of genome evolution in pinnipeds is about one rearrangement per 10 million years. This corresponds to ancestral, or slow, pattern of eutherian genome rearrangements [29].

## Conclusions

Comparative chromosome maps constructed for species from all three pinniped families (Otariidae, Odobenidae and Phocidae) with human, dog and stone marten painting probes verify G-banding data confirming hypothesis about low rate of karyotype evolution in pinnipeds. Our data demonstrate that only few tandem fusions and apparently no major inversions took place during formation of pinniped karyotypes. Phylogenetically comparative chromosome painting provides chromosomal signature for Odobenidae/Otariidae affinity. These results close a lacuna in painting data for two pinniped families and corroborate knowledge base about genome rearrangements of Carnivora as far as of all Eutheria providing strong support for presented version of Ancestral Carnivore Karyotype with 2n = 38. Still our knowledge about chromosomal and genome evolution in pinnipeds is limited and warrants for further painting experiments for other species from Otariidae and Phocidae subfamilies. The pinniped chromosome maps presented here provide robust platform for future anchoring of sequencing assemblies to chromosomes paving the way to analyses of evolution of unique adaptations to semi-aquatic lifestyle.
